# The WIN-speller: a new intuitive auditory brain-computer interface spelling application

**DOI:** 10.3389/fnins.2015.00346

**Published:** 2015-10-06

**Authors:** Sonja C. Kleih, Andreas Herweg, Tobias Kaufmann, Pit Staiger-Sälzer, Natascha Gerstner, Andrea Kübler

**Affiliations:** ^1^Department of Psychology, University of WürzburgWürzburg, Germany; ^2^KG Jebsen Centre for Psychosis Research, Division of Mental Health and Addiction, Oslo University Hospital and Institute of Clinical Medicine, University of OsloOslo, Norway; ^3^Rehabilitationszentrum Bethesda, Beratungsstelle für Unterstützte KommunikationBad Kreuznach, Germany

**Keywords:** Brain-Computer Interface (BCI), auditory, motor-impaired end-user, P300, communication

## Abstract

The objective of this study was to test the usability of a new auditory Brain-Computer Interface (BCI) application for communication. We introduce a word based, intuitive auditory spelling paradigm the WIN-speller. In the WIN-speller letters are grouped by words, such as the word KLANG representing the letters A, G, K, L, and N. Thereby, the decoding step between perceiving a code and translating it to the stimuli it represents becomes superfluous. We tested 11 healthy volunteers and four end-users with motor impairment in the copy spelling mode. Spelling was successful with an average accuracy of 84% in the healthy sample. Three of the end-users communicated with average accuracies of 80% or higher while one user was not able to communicate reliably. Even though further evaluation is required, the WIN-speller represents a potential alternative for BCI based communication in end-users.

## Introduction

Communication based on Brain-Computer Interface (BCI) technology was shown to be possible in the visual but also in the auditory modality (Nijboer et al., 2008; Furdea et al., 2009; Halder et al., 2010; Kleih et al., 2010; Höhne et al., 2011; Schreuder et al., 2011). The P300 is commonly used as an input signal and represents a positive deflection in the EEG occurring 300 ms after the occurrence of a relevant stimulus, or target, presented within a stream of irrelevant stimuli, or non-targets (oddball paradigm, Sutton et al., 1965). In an auditory spelling paradigm the target is the desired letter presented within the non-target letters of the alphabet. By focusing attention on the target, the P300 is elicited and can be classified by the BCI system. As consecutive presentation of all letters is time consuming and potentially exhausting for the user letters can be grouped and represented by codes such as tones or visual cues. Thus, first one target code can be selected and subsequently the target letter (Furdea et al., 2009; Höhne et al., 2011; Schreuder et al., 2011; Baykara et al., 2015; Halder et al., 2015). Schreuder and colleagues integrated the letters of the alphabet into six letter groups of which five included letters only while one group represented the letter Z and the backspace key (Schreuder et al., 2011). Groups were coded by a specific combination of base tone and noise. Auditory codes were presented from one of six loudspeakers arranged in a circle around the user, thus, combining code stimuli and spatial information. Healthy participants spelled with an average accuracy of 76%.

Höhne et al. (2011) investigated an auditory paradigm in which they integrated the spatial information via headphones. Their 12 healthy volunteers spelled with an average accuracy of 78% which was similar to the results reported by Schreuder et al. (2011). However, both spelling paradigms require high mental workload as not only a mental representation of the letter groups have to be retained in memory but also of the respective code. To reduce this workload, visual support matrices can be implemented, however, the ability to control gaze is then mandatory (Furdea et al., 2009).

Most recently Höhne and Tangermann (2014) presented a streaming paradigm for auditory spelling. No visual perception is necessary as letters are presented in a constant auditory stream. The user focuses attention to the target letter within this stream. Höhne and Tangermann (2014) reported an average performance of 41% with a binary linear discriminant analysis classification in ten healthy subjects. The complexity of this paradigm might be challenging for end-users as simultaneous presentation of auditory stimuli requires intense attention allocation to target, and accuracy would need to be higher for communication (70% according to Kübler et al., 2001).

In the current study, we aimed at a practical auditory speller based on natural words as the beneficial effect of implementing natural stimuli (i.e., syllables) on BCI performance was previously shown (Höhne et al., 2012). Here we used words as stimuli to represent the letters these words contained, e.g., the word KLANG which is the German word for “sound” would represent the letter group A, G, K, L, N. No code is necessary as the stimuli are composed of the letters in the respective group, thereby presumably reducing work load as no codes but just intuitive word stimuli have to be retained in mind. To support mental representation of words, phonologically different words are preferable as those facilitate recall (Conrad and Hull, 1964). Additionally words should not contain too many syllables as recall performance drops drastically from 90% in monosyllables to 50% in five syllable words (Baddeley et al., 1975). Nonetheless, using an auditory BCI system is a demanding task as compared to visual BCIs even if the stimuli are carefully chosen (Nijboer et al., 2008; Kübler et al., 2009): Thus, we were interested in intra-individual psychological variables possibly influencing BCI performance such as a person's allocated attention, the self-efficacy belief (Bandura, 1977, 1997) and the tendency for approach or avoidance behavior (Gray, 1972, 1987).

It is known that attention allocation increases the P300 amplitude in an oddball paradigm (e.g., Johnson, 1986; Polich, 1986). More recently, attention was also investigated in a P300 based BCI paradigm and the ability to filter information actively during BCI use was identified as an influencing variable in a visual BCI spelling paradigm (Riccio et al., 2013). Therefore, we suggest that attention allocation does also influence the P300 amplitude in an auditory BCI spelling paradigm and should be controlled for with an attention test.

The self-efficacy belief represents a person's expectancy of success or the belief of being able to perform well (Bandura, 1977). As pointed out by Cleary and Zimmerman (2001), there is a plethora of proof that self-efficacy beliefs and academic achievement are related (e.g., Lent et al., 1984; Zimmerman, 1990, 2000). People who score high on self-efficacy tend to set goals more specific while those who are not convinced of being able to successfully master a task set vague goals which prevent proper evaluation at the end (Cleary and Zimmerman, 2001, 2004). When using a BCI system it may be that participants with high self-efficacy beliefs perform better as they form a clear representation of the goal to be achieved.

Another psychological variable which may influence BCI users, is their behavioral orientation toward approach or avoidance (Gray, 1972, 1987). Participants whose Behavior-Inhibition System (BIS) is more dominant, might react less positive to the experience of spelling correctly as compared to participants whose Behavior-Activation System (BAS; Carver and White, 1994) is dominant and who are very sensitive to positive and rewarding experiences. While in the BIS the right prefrontal cortex (PFC) is highly activated leading to sensibility for punishment and avoidance-oriented behavior, in the BAS left prefrontal activation is increased resulting in approach-oriented behavior. As Brain-Computer Interface technology for spelling is probably unfamiliar to most participants and success cannot be estimated easily, BCI use might be more attractive for BAS users as compared to BIS users who might fear failure.

To summarize, the goals of this study were: (1) to present an easy to use, intuitive auditory spelling paradigm which is independent from visual input and allows for reliable communication, (2) to validate this paradigm in motor-impaired end-users who are the target population of auditory BCI research, and (3) to investigate possible relations between attention, self-efficacy belief, and approach-avoidance behavior with BCI performance.

We hypothesized that with the here presented auditory speller, meaningful communication with accuracies of at least 80% can be achieved (H1a) and that this 80% accuracy would be reachable with the same number of sequences as compared to a visual spelling paradigm (H1b). Amendatory to this hypothesis, we believe that higher ability for mental representation, or memory performance leads to higher spelling accuracy (H2). We hypothesized that participants who are highly attentive will show higher P300 amplitudes in the auditory paradigm as compared to participants who are less attentive (H3). Furthermore, we predicted that participants with high self-efficacy belief outperform participants with low self-efficacy belief in terms of spelling accuracy (H4). Finally, we hypothesized participants who score higher on BAS to outperform participants who score higher on BIS with respect to spelling accuracy and P300 amplitude (H5).

## Methods and materials

### Participants

We included *N* = 11 healthy participants (age *M* = 23.64, *SD* = 3.61, two male) in the study and four end-users with motor-impairment. An additional *N* = 2 subjects (one male, 27 and one female, 32) performed a free-spelling session as a proof of principle. All healthy participants were naïve to BCI use and none of them reported a history of neurological or psychiatric disease. An overview of the end-users is provided in Table 1. All end-users were male and able to communicate either by voice (*N* = 1) or by assistive technology for communication (*N* = 3). End-user A used a joystick based communication device but could also whisper sounds which can be translated to language by people who know the patient. End-user B used a voice translator which translated the words detected on the larynx into words that can be heard and understood. End-user C used a joystick based technology but most often relied on his caregiver who knows him for years and can translate his expressions to language. End-user D was not yet in need of assistive technology or caregivers for communication. We categorized the level of impairment as suggested by Kübler and Birbaumer (2008). The category *minor* indicates only slight impairment but normal speech, while *moderate* refers to patients who are in need of a wheelchair but speech is unaffected. Patients who are tetraplegic with restricted speech are categorized as *majorly* impaired. All end-users had normal or corrected to normal vision. All participants received a monetary reimbursement of 8 Euros per hour and gave informed consent to the procedure which was approved by the Ethical Review Board of the Medical Faculty, Eberhard-Karls-Univerity of Tübingen. If necessary, the legal representatives gave written informed consent (for end-users B and C).

**Table 1:** **End-user participant description**.

**End-user**	**Age**	**Diagnosis**	**Level of impairment**	**Year of diagnosis**
A	43	Traumatic accident	moderate	2004
B	49	Muscle dystrophy (Duchenne)	major	1972
C	58	Traumatic accident	major	2000
D	72	Amyotrophic Lateral Sclerosis	minor	2012

### Questionnaires

Questionnaire data was only assessed from healthy subjects not from end-user participants. To assess verbal learning ability and memory, we used the verbal learning and memory test (“Verbaler Lern-und Merkfähigkeitstest” VLMT, Helmstaedter and Durwen, 1990). A 15 items word list is read out loud to the participant who has to recall as many words as possible. This procedure is repeated five times. For measurement of delayed retrieval ability, participants recall the word list after 30 min. Recognition ability is assessed by presenting a word pool of 35 words of which the participant indicates which words belong to the word list. Memory performance parameters are *learning, consolidation* and *recognition* and were T-score normed (*M* = 50, *SD* = 10).

Attention was assessed with the d2-test (Brickenkamp, 1994). In this paper–pencil test, target stimuli (the letter d with two dashes) and non-target stimuli (the letter d with more or less than two dashes and the letter p) have to be discriminated (marking of target stimuli only) while pressed for time. The attention parameters are *general performance* and *concentration* and were evaluated using the percentile rank.

The self-efficacy belief was assessed with the generalized self-efficacy scale (GSES; Schwarzer and Jerusalem, 1995) which measures the belief of being able to master challenging situations as well as trust in one's own ability (e.g., “I am confident that I could deal efficiently with unexpected events”). The 10 items have to be rated on a four point Likert scale (1 = not at all true to 4 = exactly true) and were T-score normed (*M* = 50, *SD* = 10).

To assess approach and avoidance behavior, we used the BIS/BAS-Scales (Carver and White, 1994), which comprise 24 items on four scales: BAS *drive* (e.g., “I go out of my way to get things I want”), BAS *fun seeking* (e.g., “I crave excitement and new sensations”), BAS *reward responsiveness* (e.g., “When I get something I want I feel excited and energized”), BIS (e.g., “Criticism of scolding hurts me quite a bit”) and four filler items. The items are rated on a four point Likert scale (1 = very true for me to 4 = very false for me).

With a custom-made post-test questionnaire, we asked participants to rate their perceived level of difficulty and their required concentration on a visual analog scale ranging from 0 to 10. In open questions, we asked for strategies they used and possible explanations for making mistakes. Finally, we invited the participants to suggest improvements for the spelling application.

### BCI spelling paradigms and stimulus material

To compare auditory and visual presentation modalities, an auditory, a visual, and a multimodal paradigm including both modalities were presented to the participants. Stimulus words were “MOPS,” “BUCH,” “KLANG,” “FEDER,” “WITZ,” and the non-word “JQVXY.” Stimulus words contained all letters of the alphabet, were phonetically diverse and contained at most two syllables. Furthermore, all but one word were meaningful German words which can easily be remembered: “MOPS” = a pug dog, “BUCH” = book, “KLANG” = sound, “FEDER” = feather, “WITZ” = joke, “JQVXY” = non-word. Therefore, we created a **w**ord based **in**tuitive auditory paradigm: the WIN-Speller. Word stimuli and letters, as well as instructions (“please focus on the word ‘BUCH’ now”) were recorded by a female voice using a TBone microphone and the Cubase LE5 Software and were normalized for auditory presentation. Word stimuli ranged between 401 and 1162 ms in duration and the inter-stimulus-interval was 200 ms. During system calibration, word stimuli were presented in random order and the participant focused attention on the target word containing the target letter. Every word stimulus was a target stimulus once during calibration and was presented with a likelihood of 16.67%. After the selection of a word, the single letters and a “back” option for correction of erroneous selections were presented. Again, the participant had to focus attention on the target.

**Figure 1:**
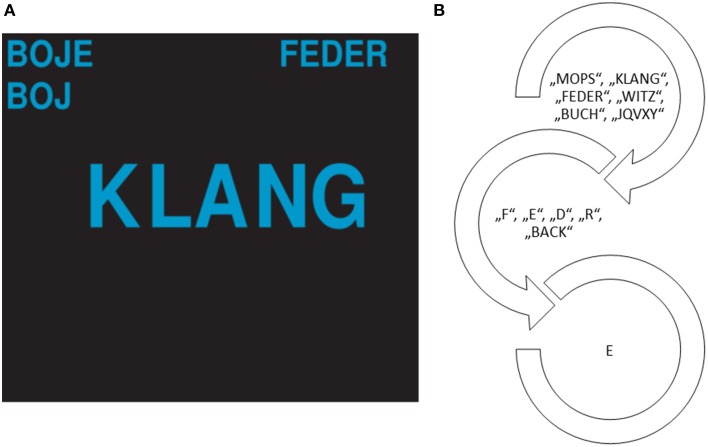
**The presentation and feedback screen in the visual paradigm (A) and the presentation in the auditory paradigm (B)**. The word BOJE had to be spelled and the next target letter is E so the target stimulus word is FEDER.

In the visual and the multimodal paradigm, the ISIs were 200 ms. The word to spell and the stimuli words were presented on the screen with a duration of 125 ms and selected letters appeared on the top left margin of the screen below the target word display (see Figure 1A). In the auditory paradigm, the WIN-speller, stimulation as well as feedback were purely auditory (see Figure 1B). The word to spell was presented to the participant via headphones (“please spell now the word ‘BOJE”’). Then the target code word was read to the participant (“please focus now on the code word ‘BUCH”’ to spell the letter “B”). After successful choice of the code, the target letter was announced (“you chose the word ‘BUCH,”’ now focus on the letter “B”). Feedback about letter selection was provided and updated after every letter selection (“You just chose the letter “J.”' So far you spelled “BOJ”).

### Information transfer rate (ITR)

We calculated the ITR as bits per minute (B) including information on accuracy and number of possible outputs (1):

(1) $$\begin{array}{l} B={log}_{2}N+Pl{og}_{2}P+\left(1-P\right){log}_{2}\left[\left(1-P\right)∕\left(N-1\right)\right] \end{array}$$

*N* is the number or possible outputs, *P* is the probability that the desired selection is produced with all possible selections having the same probability of being produced (Shannon and Weaver, 1964; Pierce, 1980).

### Procedure in healthy participants

All questionnaires were presented to the participants prior to the BCI measurement. Auditory, visual, and multimodal paradigms were counterbalanced across subjects. To avoid fatig, we presented only one spelling paradigm in the first session (day 1) and the other two in the second session (day 2).

The BCI was calibrated separately for the visual, the auditory and the multimodal modality prior to each paradigm. For the WIN-speller, the six stimulus words were presented to participants via headphones in randomized order while they had to focus on one of the words. In the multimodal paradigm, the auditory presentation of stimuli was complemented by a visual display of the words. In the visual paradigm participants had to focus attention to one predefined word appearing in the center of the screen. All word stimuli were presented for 10 sequences which equals a repetition of 20 times per word stimulus in all modalities. Number of sequences to spell 80% correct were determined for each individual. During calibration no feedback was provided to the participants. After calibration, participants had to copy-spell (Kübler et al., 2001) the words “BOJE,” “SYLT,” and “HARZ.” We chose these words because to spell them each stimulus word had to be selected at least once while avoiding duplication of the target letter.

We additionally assessed data of two healthy volunteers only using the WIN-speller in free spelling mode as a proof of principle of this paradigm. In this free spelling, the participants could freely choose what to spell and thus, were not supported by the system by instructions on which stimulus to focus on.

### Procedure in end-users

The four end-users who participated in this study, only tested the WIN-speller in one session in the auditory paradigm as this was the spelling paradigm to be validated with them. They also spelled the words “BOJE,” “SYLT,” and “HARZ” but did not receive the word stimuli prior to the session. We did not provide them with the words before testing because we were interested whether the system could be used successfully by end-users also in case they do not know the target words before having to use them. For stimulus presentation in end-users we used loudspeakers as we were interested in the applicability of the paradigm even in cases in which headphone positioning might be impossible.

### Data acquisition

Stimulus presentation was implemented in Python© (version 2.5, Python Software Foundation) and linked via UDP to BCI2000 (version 3, Schalk et al., 2004), which was used for data recording and storage. EEG was measured with an electrode cap (easy cap) with 12 Ag/AgCl electrodes located at positions F3, Fz, F4, C3, Cz, C4, P3, Pz, P4, PO7, PO8, and Oz following the international 10–10 standard system (American Electroencephalographic Society, 1994) referenced to the right and grounded to the left mastoid. Data was filtered online with a high pass of 0.1 Hz, a low pass of 30 Hz and a notch filter at 50 Hz. The EEG signal was amplified with a g.USBamp (Guger Technologies, Austria). Impedance was kept below 5 kΩ and the sampling rate was 256 Hz. Data processing, storage and stimulus presentation was controlled with a computer (Intel Core 2 Duo, 4 GHz, Windows 7), loudspeakers were Hama AL-140 Stereo Speaker (Monheim, Germany). For data classification online and offline stepwise linear discriminant analysis (SWLDA) was applied (for details see e.g., Krusienski et al., 2008). All electrodes were included to calculate the feature weights on which the classification was based.

### Data analysis

For offline P300 analysis in healthy subjects, EEG data were corrected for artifacts (>70 μV) and baseline (–100 to 0 ms) using MATLAB© (v2011b). Trials, which included artifacts, were excluded from further analysis, which applied to < 5% of all data. The P300 was defined as the maximum positive peak between 200 and 600 ms after stimulus onset identified by semiautomatic global peak detection using MATLAB© (v2011b). Semiautomatic peak detection suggest the global highest peak within the predefined time frame of 200 to 600 ms, but the chosen peak has to be confirmed by the user to accept the value as P300 amplitude value for further analysis. Targets and non-targets were averaged and grand averages were contrasted for the three spelling paradigms. Dependent variables were spelling accuracy as measured in percent of correctly spelled characters, required sequences to reach an accuracy of at least 80%, P300 amplitude and latency. For correlations, we used Bonferroni correction for multiple comparisons. The level of significance was set to α = 0.05 and IBM SPSS 20® was used for statistical analysis.

## Results

### Performance

With an average online accuracy of 83.69% (*SD* = 20.73, see Table 2) in healthy volunteers H1a was confirmed. Only three of eleven participants could not reach 70% accuracy which is the minimum accuracy required for communication (Kübler et al., 2001) while eight participants spelled with above 90% accuracy.

**Table 2:** **Online spelling accuracies in the auditory paradigm**.

	**BOJE**	**SYLT**	**HARZ**	***M***	***SD***
VP1	90	83.33	100	91.11	8.39
VP2	50	16.67	62.50	43.06	23.69
VP3	100	87.50	100	95.83	7.22
VP4	40	80	75	65	21.79
VP5	100	100	100	100	0
VP6	100	100	100	100	0
VP7	90	100	83.33	91.11	8.39
VP8	100	100	100	100	0
VP9	100	90	100	96.67	5.77
VP10	100	90	90	93.33	5.77
VP11	41.67	10	100	50.56	45.65
*M*	82.88	77.95	91.89		
*SD*	25.45	32.76	12.99		

**Figure 2:**
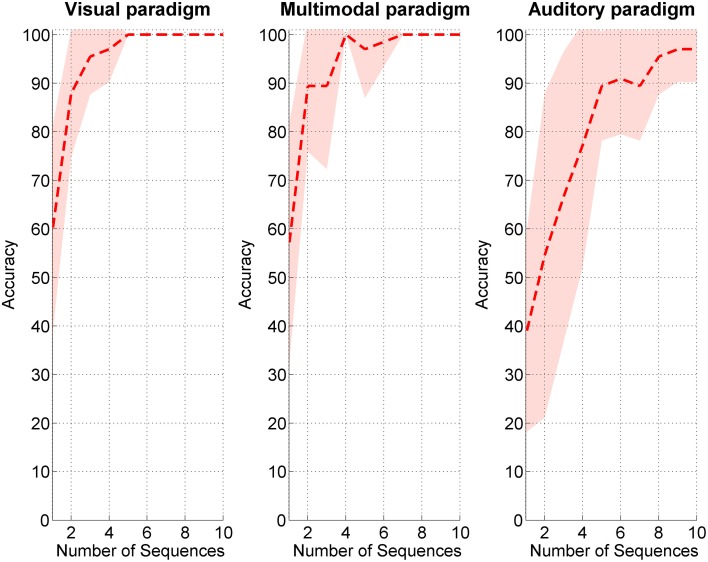
**Sequences needed to spell 80% correct in the three spelling paradigms**. Standard deviation is depicted in light red.

Average accuracies with the visual and multimodal paradigms were higher than with the auditory (*M*_*visual*_ = 97.73%, *SD* = 4.73; *M*_multimodal_ = 92.68%, *SD* = 10.40), but a Three-Way repeated measures ANOVA with *modality* as within subject factor and *accuracy* as dependent variable yielded no significant differences between the paradigms [*F*_(2, 20)_ = 3.26, *p* = 0.06]. When comparing the number of required sequences to reach an accuracy of at least 80% (see Figure 2), Three-Way repeated measures ANOVA with *modality* as within subject factor and *sequences* as dependent variable yielded significant differences between the paradigms [*F*_(2, 20)_ = 10.64, *p* = 0.001]. In the visual paradigm participants needed significantly less sequences (*M* = 4.73, *SD* = 1.27) to achieve 80% accuracy as compared to in the WIN-speller {*M* = 8.0; *SD* = 2.41, *post hoc* contrast [*F*_(1, 10)_ = 18.36, *p* = 0.002]} but no difference as compared to the multimodal speller was found [*M* = 5.08, *SD* = 1.50, *post hoc* contrast *F*_(1, 10)_ = 3.45, *p* = 0.09].

We also compared the Information Transfer Rates in the three paradigms using Three-Way repeated measures ANOVA and found a significant difference between modalities [*F*_(2, 20)_= 6.24, *p* = 0.008]. *Post hoc* comparisons revealed that the WIN-speller ITR was significantly lower (*M* = 1.11, *SD* = 0.71) as compared to the visual modality ITR [*F*_(1, 10)_ = 12.57, *p* = 0.005, (*M* = 2.04, *SD* = 0.68)] but only marginally different from multimodal paradigm ITR [*F*_(1, 10)_ = 4.71, *p* = 0.06, (*M* = 1.70, *SD* = 0.70)]. Therefore, H1b could not be confirmed by the here presented data.

**Figure 3:**
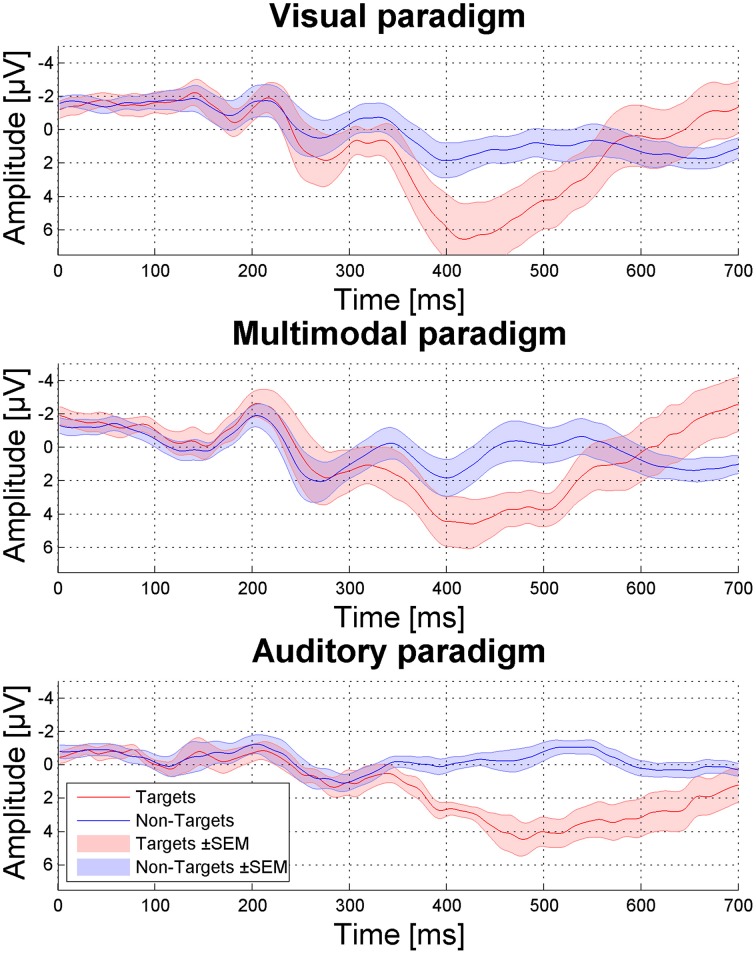
**P300 amplitudes for the three spelling paradigms depicted from Cz**. Red and blue shades indicate the standard error of the mean (SEM).

P300 amplitudes on Cz did not significantly differ between paradigms [*F*_(2, 10)_ = 0.94, *p* = 0.12, see Figure 3, *M*_visual_ = 9.38, *SD* = 5.86; *M*_multi_ = 8.56, *SD* = 5.83; *M*_win_= 6.27, *SD* = 3.20] as tested with Three-Way repeated measures ANOVA (*modality* as factor and *P300 amplitude* as dependent variables).

### The effect of memory on performance

Hypothesis H2 stated better memory to positively affect BCI performance when using the WIN-speller. The outcome parameters of the VLMT *learning, consolidation*, and *recognition* (see Table 3) yielded high scores on average. However, correlation calculation between these outcome parameters and accuracy were not significant.

**Table 3:** **Test values for the VLMT (***T***-values) and the d2-test (percentile ranks) subscales**.

	**VLMT learning**	**VLMT consolidation**	**VLMT recognition**	**D2 concentration**	**D2 overall performance**
VP1	67	66	54	98	99
VP2	67	64	54	99	99
VP3	67	63	54	79	86
VP4	67	63	54	99	99
VP5	67	63	53	99	99
VP6	67	63	54	82	87
VP7	67	59	54	99	99
VP8	66	64	54	99	99
VP9	67	59	54	97	99
VP10	67	63	54	66	72
VP11	66	63	54	99	99
*M*	66.82	62.77	53.91	92.36	94.27
*SD*	0.40	2.09	0.40	11.40	8.92

### The effect of attention on the P300 amplitude

Our third hypothesis stated that highly attentive participants should show higher P300 amplitudes. When correlating the attention parameters *d2 concentration* and *d2 overall performance* (see Table 3) with the P300 amplitudes on Cz, we did not find significant correlations. Therefore, our second hypothesis was rejected.

### The effect of self-efficacy beliefs on performance

To address our fourth hypothesis of participants with strong self-efficacy beliefs to perform better in the WIN-speller paradigm, we correlated the GSES total (see Table 4) with achieved accuracies and found no significant correlations. We rejected H4.

**Table 4:** **Test values for the GSES and the BIS/BAS subscales**.

	**GSES**	**Behavior inhibition**	**Behavior activation *drive***	**Behavior activation *fun seeking***	**Behavior activation *reward responsiveness***
VP1	30	2.71	3.25	3	3.6
VP2	28	3.29	3	3.25	3.6
VP3	30	1.71	3.75	2.75	2.6
VP4	29	3.14	1.75	3.75	3.4
VP5	31	2.43	3.75	2.75	3.4
VP6	30	2.29	3	3	3
VP7	26	4	2.75	2.5	2.75
VP8	32	2.86	3	3	3.5
VP9	30	2.43	2.5	2.75	2.75
VP10	28	2.86	2.5	3.25	2.5
VP11	28	3.71	2.75	3.25	3
*M*	29.27	2.86	2.91	3.02	3.02
*SD*	1.68	.57	.57	.34	.34

### The effect of behavior activation or behavior inhibition orientation on performance

Our fifth hypothesis predicted that participants who score high on BAS should achieve higher accuracies in the WIN-speller as compared to participants who score high in BIS (*M* = 2.86, *SD* = 0.66). We found no significant correlation between BIS and accuracy. We also correlated the P300 amplitude with the three subscales BAS *drive*, BAS *fun seeking*, and BAS *reward responsiveness* (see Table 4), using Spearman's rho and found no significant correlations. Therefore, H5 was rejected.

### Results of the custom-made posttest questionnaire

Participants judged the WIN-speller to be more difficult (*M* = 6.35, *SD* = 2.25, ranging from 0 to 10) as compared to the visual (*M* = 2.84, *SD* = 2.53) or the multimodal paradigm (*M* = 3.81, *SD* = 2.59). Participants reported that focusing attention was easiest using the WIN-speller (*M* = 1.45, *SD* = 1.86) as compared to the visual (*M* = 2.10, *SD* = 1.76) or multimodal paradigm (*M* = 2.25, *SD* = 2.73). Discriminability of stimuli was judged to be highest for the WIN-speller (*M* = 1.47, *SD* = 1.94) and the multimodal paradigm (*M* = 1.57, *SD* = 1.19) as compared to visual stimuli (*M* = 2.30, *SD* = 2.28).

Concerning strategies for attention allocation, six participants reported counting the number of times the stimuli were presented. Two participants imagined the stimulus words as pictures (a pug dog when the word “MOPS” was presented). Two participants stated that they had imagined the stimulus words to flash up in their minds. Two participants focused on the sound of the words and another two tried to complete the words in their mind while listening to the first letters being pronounced.

As possible reasons for errors, five participants reported decreased concentration with time while three participants reported distracting thoughts while using the auditory paradigm or having forgotten on which word to focus. Participants suggested that using different speakers for stimuli recording might increase stimulus discriminability. Furthermore, it was difficult to focus on an auditory paradigm with eyes open (three participants). One participant criticized the length of the paradigm.

### Free spelling mode as a proof of principle

Both healthy volunteers successfully used the WIN-speller paradigm. The first participant reached 86% accuracy when writing the sentence “it is very warm in here ice cream.” The second participant spelled the sentence “the fox laughs” with 82% accuracy. Calibration predicted accuracies of 100% when using two sequences, which was the number of sequences chosen for the free spelling.

### Validation of the WIN-speller paradigm in motor-impaired end-users

**Figure 4:**
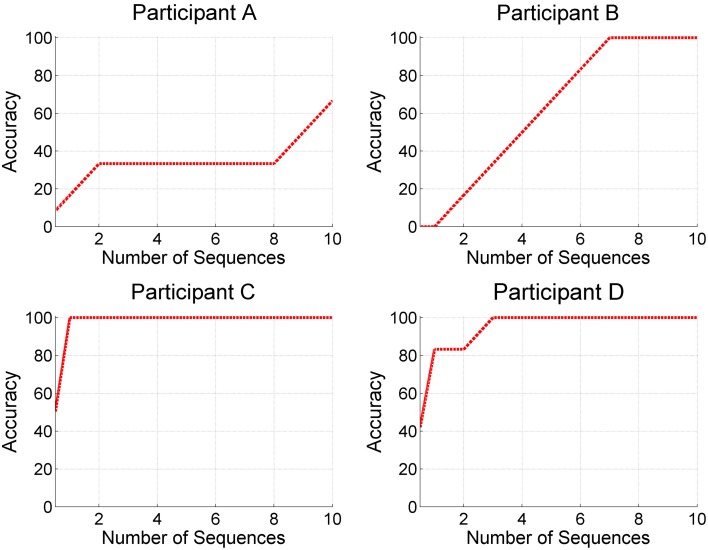
**Accuracies reached by motor-impaired end-user participants and according required number of sequences**.

Three end-user participants (B, C, and D) achieved average online accuracies of 84.17% (range 75–100%), 80% (range 50–100%), and 80.83% (range 62.5–100%, see Figure 4). Participant A achieved an average online accuracy of 51.85% (range 50–55.56%, see Figure 4).

**Figure 5:**
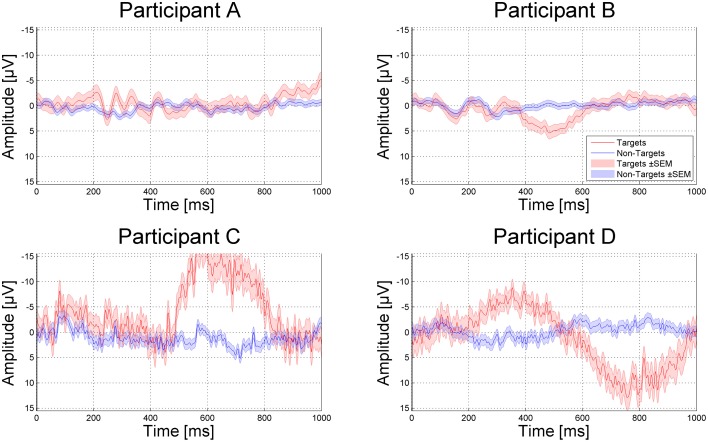
**P300 evoked by targets (red) compared to non-targets (blue) depicted from Cz**. Red and blue shades indicate the standard error of the mean (SEM).

While in participant B, the P300 was very clearly detectable (see Figure 5), targets and non-targets were less distinguishable in participant A (see Figure 5). In participant C immense muscle spasms were triggered by the target stimulus presentation. These muscle spasms caused heavy artifacts but did not hinder the patient from selecting the target letters correctly. However, it might be possible that indeed the muscle spasms were classified instead of the target ERP response. The Information Transfer Rate (ITR) was 0.28 for participant A, 1.14 for participant B, 2.13 for participant C, and 1.77 for participant D.

### Results of the custom-made posttest questionnaire

As end-users reported to feel exhausted after finalization of the spelling task, we interviewed them about their experience with the WIN-speller. This interview was based on the same custom-made posttest questionnaire as for the healthy participants but data was assessed by report instead of writing. However, we only assessed the questions about the auditory paradigm as the other modalities were not assessed with end-users. Participants B and D reported that they were surprised how well the WIN-speller worked but that they would prefer a faster presentation of the stimuli. Participant A clearly stated that he almost could not wait for the next stimulus to be presented and that he thought the paradigm was much too slow. He assumed this long presentation time to have caused his errors. Participant C reported that he was very happy to see that he could control the system and might be able to use it for communication. He also tried another auditory spelling paradigm (Baykara et al., 2015) and was not able to successfully spell letters (Halder, personal communication). None of the participants reported a strategy how to pay attention to the presented stimuli or of imagining the words as pictures. Participant D reported that he would have preferred a pronunciation of the words that is very clear and almost exaggerated to facilitate understanding even though that would possibly result in non-naturally pronounced words.

## Discussion

### Usability of the here presented WIN-speller

Healthy participants as well as end-users with motor impairment could successfully use the WIN-speller. The accuracies reached were comparable to visual spellers (e.g., Kleih et al., 2010; Liu et al., 2011; Treder et al., 2011; Kaufmann et al., 2013). Importantly, the WIN-speller is independent of visual support, however, at the cost of information transfer. The ITRs are at the lower range as compared to those reported in the literature (see Table 5). However, all studies listed in Table 3 included healthy subjects only.

**Table 5:** **ITRs achieved in auditory paradigms and the corresponding authors**.

**ITR**	**Authors**
1.54	Furdea et al., 2009
2.46	Halder et al., 2010
2.0	Klobassa et al., 2009
3.4	Höhne et al., 2010
5.26	Schreuder et al., 2011
1.11	Kleih et al., this work

We argue that in end-users with severe motor impairment accuracy might be more important than the speed with which the information can be conveyed. Furthermore, some end-users may not be able to use a multiclass spelling paradigm in which letters are coded by sounds or other stimuli because such tasks are cognitively more demanding. For end-users with motor impairment, the WIN-speller may be more intuitive and easy to use. However, we did not assess workload and our end-user sample consisted of volunteers who were not visually impaired. Furthermore, we only assessed free spelling as a proof of principle in two healthy subjects. Even though both were highly successful and fast when using the WIN-speller, the number of subjects needs to be increased to finally draw conclusions about its usability. Additionally free spelling from the end-users would have provided a good indicator of the usability of the system and would have shown us whether end-users enjoy the application for real communication.

In end-user C the presentation of the target stimuli caused muscle artifacts. These artifacts, which occasionally also occurred as a reaction to non-targets, did not hinder the participant from spelling correctly. At the same time, it might be that the BCI classified the muscle spasms instead of the EEG activity as artifacts heavily influenced all EEG traces.

Because of these limitations, we present a first proof-of-principle here. Further evaluation in healthy subjects and motor-impaired end-users is needed to finally judge the usability of the WIN-speller paradigm for the target population.

### Words as stimuli

In the WIN-speller we chose German words as stimuli. The ideal choice of words, should however, be subject of future research. It has been demonstrated that words occurring frequently in the spoken language elicit smaller P300 amplitudes as compared to infrequent words (e.g., Rugg, 1990; Hauk and Pulvermüller, 2004). Frequent activation of word representations increases neuronal connectivity and therefore requires less activation resulting in smaller P300 amplitudes (Hauk and Pulvermüller, 2004). However, contradictory results showing higher P300 amplitudes in response to frequent words, were also found (Polich and Donchin, 1988; Scott et al., 2009). Authors of these studies suggest that regular use of a word leads to facilitated and higher activation as measured with the P300 amplitude. Therefore, word frequency of in the spoken language of stimulus words used in the WIN-Speller paradigm is a variable to be thoroughly investigated in the future. Furthermore, the inclusion of speakers of both sexes as well as inclusion of spatial information (Schreuder et al., 2010) might increase discriminability of word stimuli.

### Psychological variables influencing BCI performance

We hypothesized (H2) that memory influences accuracy but found no indices for this assumption. This result together with the fact that motor-impaired end-users successfully operated the WIN-speller without previous information about the word stimuli, emphasizes the usability of the WIN-speller paradigm.

Our assumption of higher attention leading to higher P300 amplitudes (H3) was not confirmed. It might be that the d2-test used here was not suitable for investigation of our hypothesis. The d2-test is a visual attention test. Using an auditory attention test, such as the Auditory Continuous Performance Test (Keith, 1994) might have been more appropriate. Overall, the used tests might not have been sensitive enough to detect differences in healthy participants. Possibly more appropriate tests should be identified and implemented in future studies, specifically aiming at multimodal stimulus presentation and test norms for young healthy adults. Pilot studies might be necessary to investigate whether the chosen tests are sensitive enough to potentially explain variance in BCI performance.

Also the hypothesis that self-efficacy (Bandura, 1977) would influence performance was not supported by the data. It might well be that setting the goal of spelling a whole word correctly is not that much vaguer as compared to spelling a letter correctly. Therefore, goal setting strategies might not be applicable in context of the current study. Furthermore, as our healthy volunteers received monetary reimbursement, their primary goal might have been participation to receive money instead of reaching a high level of performance.

Finally we hypothesized participants who score high on BAS and to show higher P300 amplitudes than those who score high on BIS (Gray, 1972; Carver and White, 1994). We did not find users who score high on BAS to achieve higher accuracies nor higher P300 amplitudes. However, it was previously reported by Nijs et al. (2007) that higher BAS scores are correlated with higher P300 amplitudes.

Overall, the WIN-speller seems to be independent of attention, memory, self-efficacy belief, and behavioral orientation as measured with respective psychological tests. This might be encouraging as specifically attention and memory may be reduced in patients in the locked-in state with impaired vision. However, the variance in the tests we assessed was rather small and it might be that other, more sensitive instruments are needed to identify possible psychological variables affecting BCI performance.

## Conclusion

We presented a new auditory spelling paradigm, the WIN-speller, which is easy to use and, most importantly, applicable with high accuracies in motor-impaired end-users. No visual support is needed to control the speller. Possibly the WIN-speller paradigm might also be usable for end-users who cannot operate other auditory multiclass spellers which impose higher working memory load as codes and goals (words to spell) have to be maintained in mind. Following the user-centered design (Kübler et al., 2014) different auditory paradigms could be presented to an individual end-user and the most successful one with respect to effectiveness, efficiency, and satisfaction could be chosen as an individualized solution. The possible benefit of using the WIN-speller has to be evaluated in the future by end-users who cannot operate visual spellers.

### Conflict of interest statement

The authors declare that the research was conducted in the absence of any commercial or financial relationships that could be construed as a potential conflict of interest.
